# Supplementary figures and images for: 14-3-3 proteins promote synaptic localization of N-methyl d-aspartate receptors (NMDARs) in mouse hippocampal and cortical neurons
Source: PLoS One. 2021 Dec 28;16(12):e0261791. doi: 10.1371/journal.pone.0261791 (PMC8714094; doi:10.1371/journal.pone.0261791)

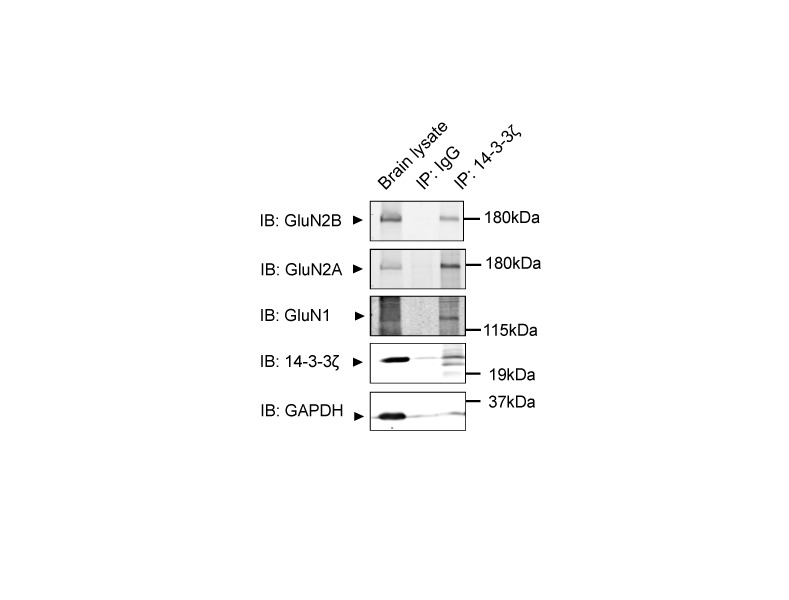

Supplement: S1 Fig — Hippocampal brain lysates from wild-type mice were immunoprecipitated (IP) with either anti-mCherry (rabbit IgG) or anti-14-3-3ζ antibodies. Hippocampal brain lysates and IP protein samples were separated by either 8% or 10% SDS/PAGE to probe for GluN1, GluN2A, and GluN2B or anti-14-3-3ζ and anti-GAPDH respectively. As a negative control, rabbit IgG were used. Images shown are representative western blots from three independent experiments. IB, antibody used for immunoblot analysis. In contrast to rabbit IgG (negative control), 14-3-3ζ coimmunoprecipitates GluN1, GluN2A, and GluN2B from hippocampal brain lysates. (TIF) [file pone.0261791.s001.tif]

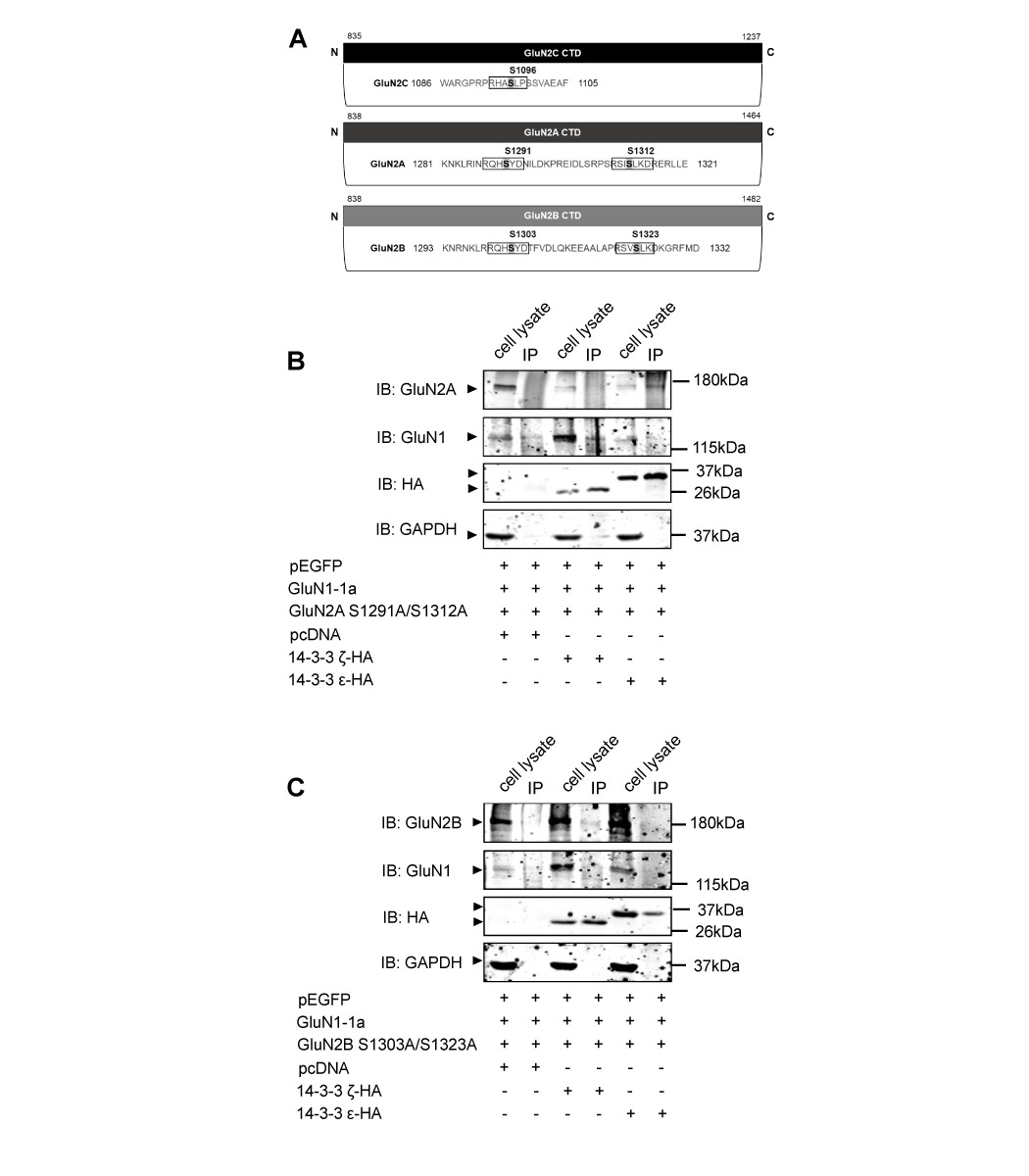

Supplement: S2 Fig — A, Potential 14-3-3 binding motifs from the C-terminal domain (CTD) region of rat GluN2A and GluN2B with known motif on rat GluN2C subunit. Predicted and known serine sites for GluN2C (S1096), GluN2A (S1291, S1312), and GluN2B (S1303, S1323) highlighted in gray and predicted binding motifs boxed in around these serine sites. B, tsA201 cells co-expressing NMDAR subunits and HA-tagged 14-3-3ζ or ε isoforms were lysed and proteins were immunoprecipitated (IP) with anti-HA antibodies. Input and IP protein samples were separated by SDS/PAGE and probed with NMDAR subunit antibodies. As controls, cell lysates expressing vector control (pcDNA) were used for comparison and all immunoblots were re-probed with anti-HA and anti-GAPDH antibodies. IB, antibody used for immunoblot analysis. 14-3-3ζ and ε coimmunoprecipitate similar levels of both GluN1 and GluN2A from cells expressing GluN2A S1291A/S1312A compared to cells expressing GluN2A wild-type (observed in Fig 6B). C, 14-3-3ζ and ε coimmunoprecipitate similar levels of both GluN1 and GluN2B from cells expressing GluN2B S1303A/S1323A compared to cells expressing GluN2B wild-type (observed in Fig 6C). (TIF) [file pone.0261791.s002.tif]
