# Supplementary material for: 14-3-3 proteins promote synaptic localization of N-methyl d-aspartate receptors (NMDARs) in mouse hippocampal and cortical neurons
Source: PLoS One. 2021 Dec 28;16(12):e0261791. doi: 10.1371/journal.pone.0261791 (PMC8714094; doi:10.1371/journal.pone.0261791)
Supplement: S1 Raw images — (PDF) [file pone.0261791.s003.pdf]

# Fig4A-1

Same blot cut below 64kDa
Top blot probed with GluN1
Bottom blot probed with GAPDH

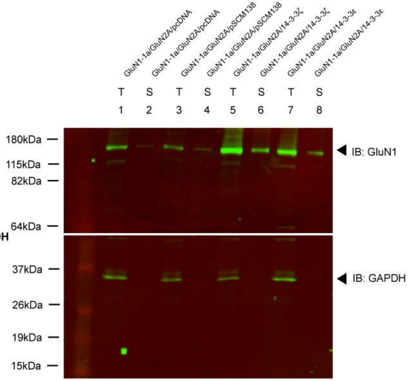


**raw file**

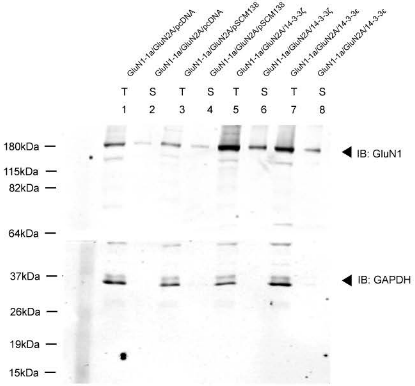


**Inverted image (grayscale)**

**Odyssey CLx Imager using Image Studio software (Ver 5.2) (LI-COR Biosciences) was used to capture all images**

**IB: immunoblot**
**T: Total proteins**
**S: Surface proteins**

## Fig4A-2

Same blot from Fig4A-1

Top blot stripped and reprobed with GluN2A

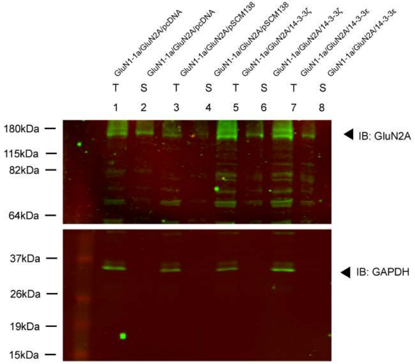


**raw file**

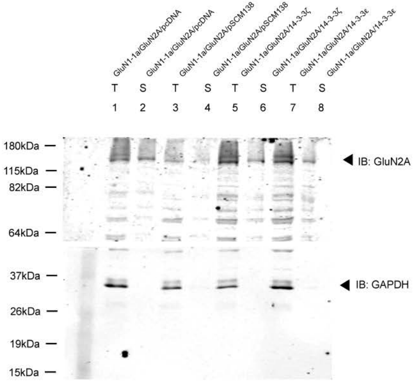


**Inverted image (grayscale)**

**Odyssey CLx Imager using Image Studio software (Ver 5.2) (LI-COR Biosciences) was used to capture all images**

**IB: immunoblot**
**T: Total proteins**
**S: Surface proteins**

## Fig4D-1

Same blot cut below 64kDa
Top blot probed with GluN1
Bottom blot probed with GAPDH

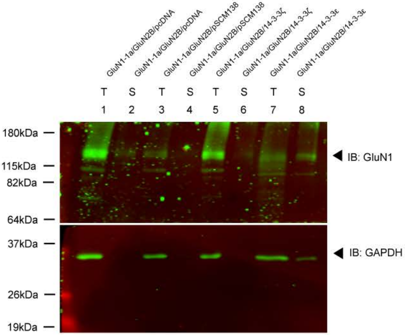


**raw file**

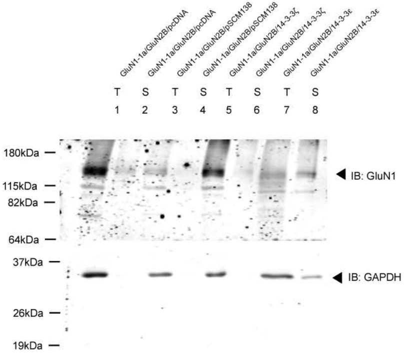


**Inverted image (grayscale)**

**Odyssey CLx Imager using Image Studio software (Ver 5.2) (LI-COR Biosciences) was used to capture all images**

**IB: immunoblot**
**T: Total proteins**
**S: Surface proteins**

## Fig4D-2

Same blot cut below 64kDa
Top blot probed with GluN2B
Bottom blot probed with GAPDH

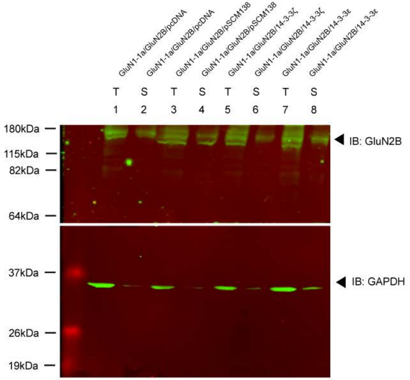


raw file

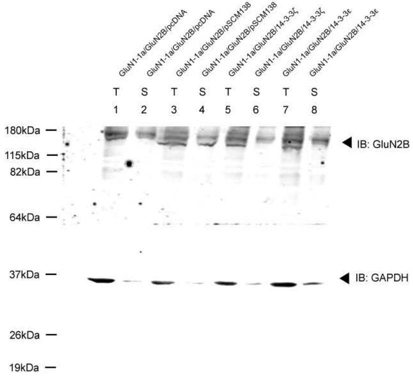


Inverted image (grayscale)

Odyssey CLx Imager using Image Studio software (Ver 5.2) (LI-COR Biosciences) was used to capture all images

IB: immunoblot
T: Total proteins
S: Surface proteins

Fig6A

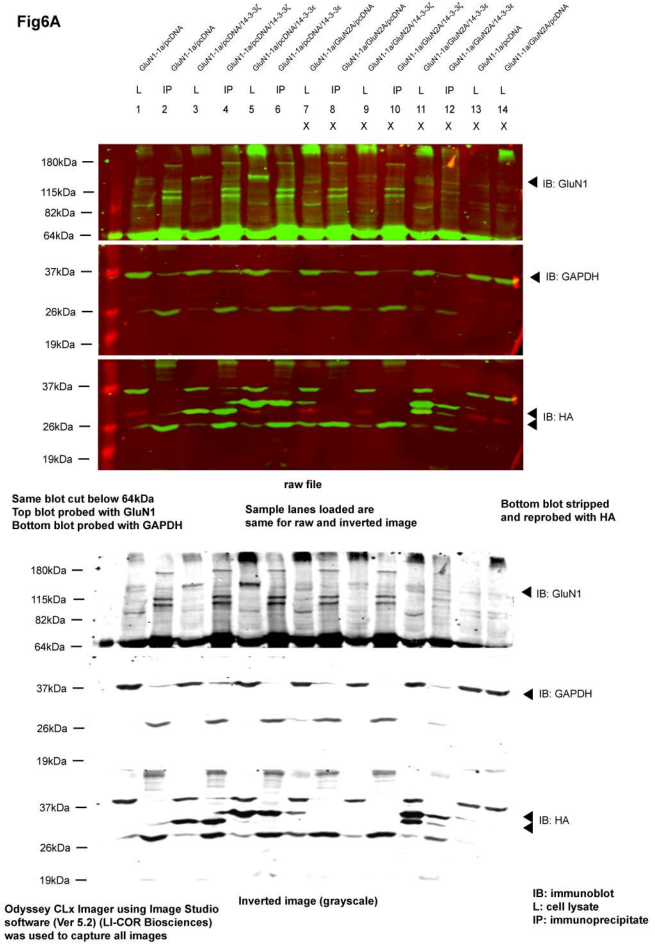
GluN1-1a/pcDNA
GluN1-1a/pcDNA
GluN1-1a/pcDNA/14-3-3ζ
GluN1-1a/pcDNA/14-3-3ζ
GluN1-1a/pcDNA/14-3-3ε
GluN1-1a/pcDNA/14-3-3ε
GluN1-1a/GluN2A/pcDNA
GluN1-1a/GluN2A/pcDNA
GluN1-1a/GluN2A/14-3-3ζ
GluN1-1a/GluN2A/14-3-3ζ
GluN1-1a/GluN2A/14-3-3ε
GluN1-1a/GluN2A/14-3-3ε
GluN1-1a/pcDNA
GluN1-1a/GluN2A/pcDNA
L IP L IP L IP L IP L IP L IP L L
1 2 3 4 5 6 7 8 9 10 11 12 13 14
X X X X X X X X
180kDa
115kDa
82kDa
64kDa
IB: GluN1
37kDa
26kDa
19kDa
IB: GAPDH
37kDa
26kDa
19kDa
IB: HA
raw file
Same blot cut below 64kDa
Top blot probed with GluN1
Bottom blot probed with GAPDH
Sample lanes loaded are
same for raw and inverted image
Bottom blot stripped
and reprobed with HA
180kDa
115kDa
82kDa
64kDa
IB: GluN1
37kDa
26kDa
19kDa
IB: GAPDH
37kDa
26kDa
19kDa
IB: HA
Inverted image (grayscale)
Odyssey CLx Imager using Image Studio
software (Ver 5.2) (LI-COR Biosciences)
was used to capture all images
IB: immunoblot
L: cell lysate
IP: immunoprecipitate

**Fig6B-1**

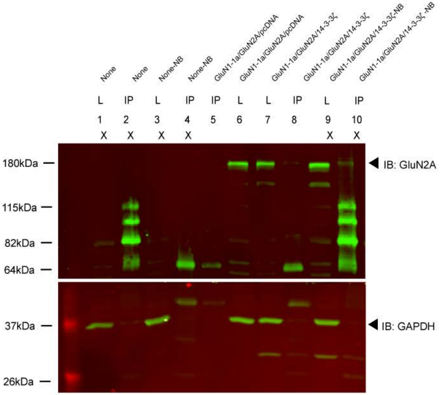


**raw file**

**Same blot cut below 64kDa**
**Top blot probed with GluN2A**
**Bottom blot probed with GAPDH**

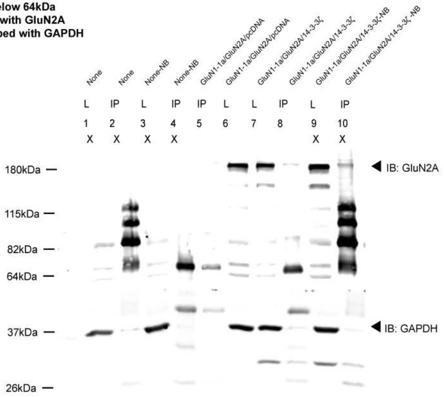


**Inverted image (grayscale)**

**Odyssey CLx Imager using Image Studio software (Ver 5.2) (LI-COR Biosciences) was used to capture all images**

**IB: immunoblot**
**L: cell lysate**
**IP: immunoprecipitate**
**NB: not boiled samples**

## Fig6B-2

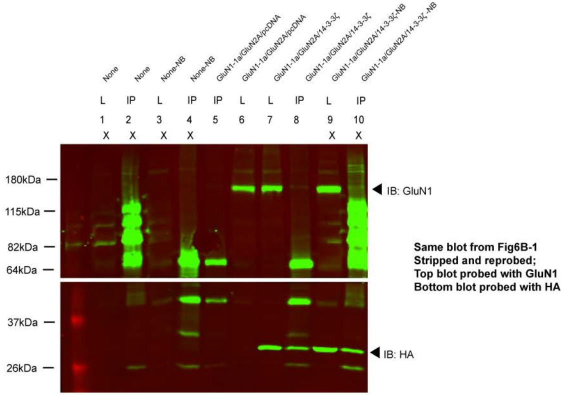


**Same blot from Fig6B-1**
**Stripped and reprobed;**
**Top blot probed with GluN1**
**Bottom blot probed with HA**

**raw file**

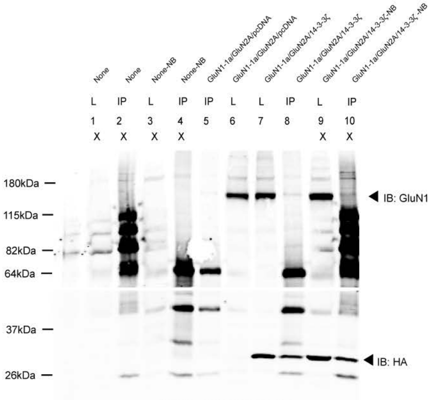


**Inverted image (grayscale)**

**Odyssey CLx Imager using Image Studio software (Ver 5.2) (LI-COR Biosciences) was used to capture all images**

**IB: immunoblot**
**L: cell lysate**
**IP: immunoprecipitate**
**NB: not boiled samples**

## Fig6B-3

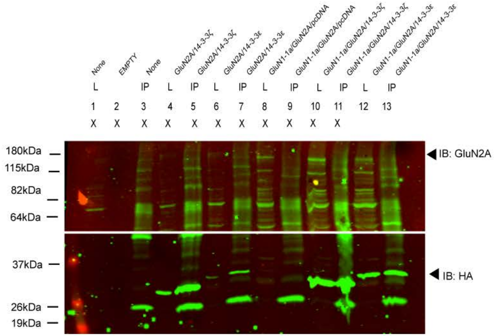


**raw file**

**Same blot cut below 64kDa**
**Top blot probed with GluN2A**
**Bottom blot probed with HA**

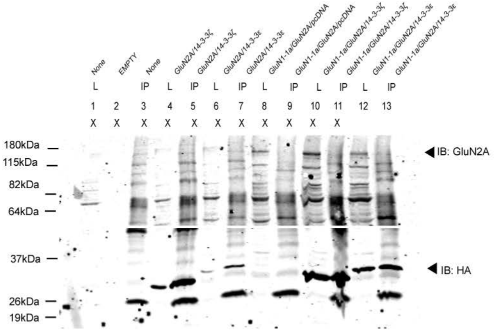


**Inverted image (grayscale)**

**Odyssey CLx Imager using Image Studio software (Ver 5.2) (LI-COR Biosciences) was used to capture all images**

**IB: immunoblot**
**L: cell lysate**
**IP: immunoprecipitate**

## Fig6B-4

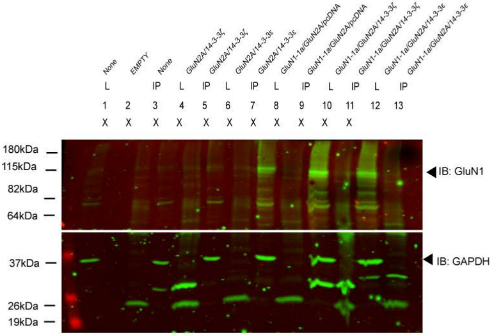


**raw file**

**Same blot as Fig6B-3**
**Stripped and reprobed;**
**Top blot probed with GluN1**
**Bottom blot probed with GAPDH**

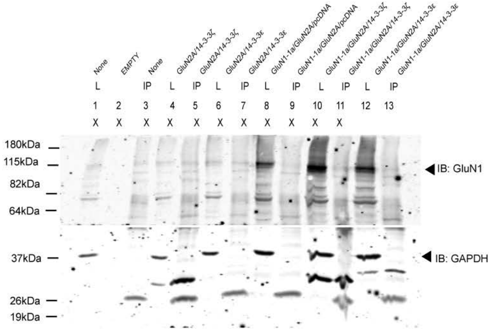


**Inverted image (grayscale)**

**Odyssey CLx Imager using Image Studio software (Ver 5.2) (LI-COR Biosciences) was used to capture all images**

**IB: immunoblot**
**L: cell lysate**
**IP: immunoprecipitate**

## Fig6C-1

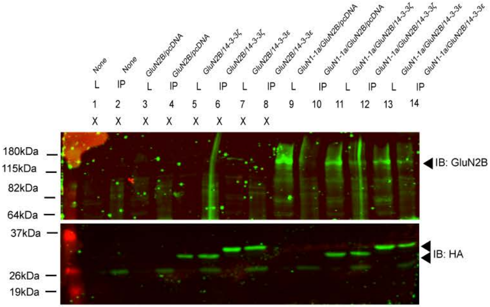


**raw file**

**Same blot cut below 64kDa**
**Top blot probed with GluN2B**
**Bottom blot probed with HA**

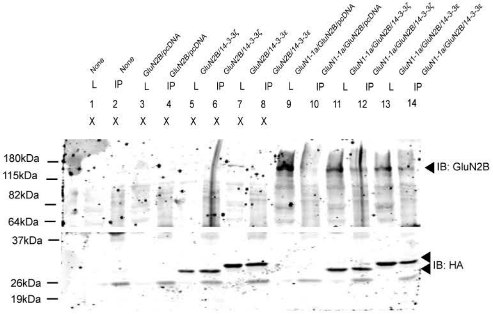


**Inverted image (grayscale)**

**Odyssey CLx Imager using Image Studio software (Ver 5.2) (LI-COR Biosciences) was used to capture all images**

**IB: immunoblot**
**L: cell lysate**
**IP: immunoprecipitate**

Fig6C-2

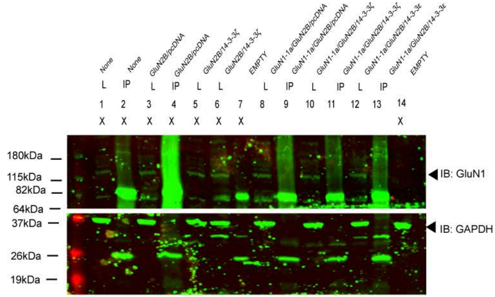


raw file

Same samples from Fig6C-1
Top blot probed with GluN1
Bottom blot probed with GAPDH

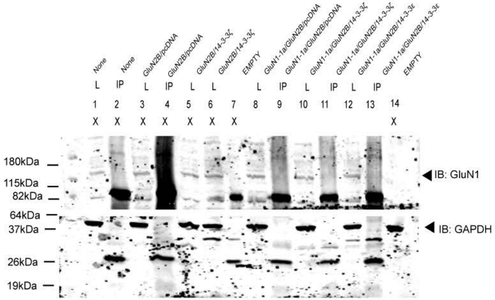


Inverted image (grayscale)

Odyssey CLx Imager using Image Studio software (Ver 5.2) (LI-COR Biosciences) was used to capture all images

IB: immunoblot
L: cell lysate
IP: immunoprecipitate

**S1 Fig-1**

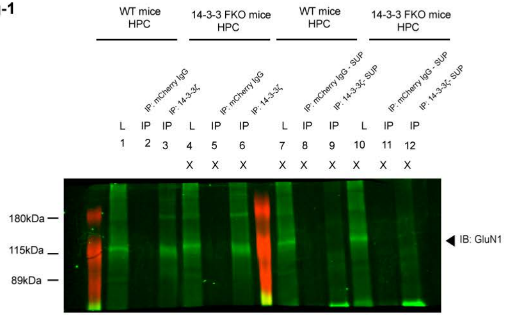


**raw file**

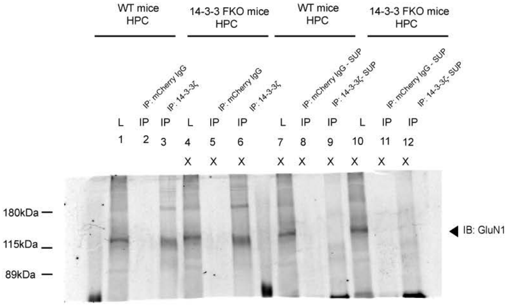


**Inverted image (grayscale)**

**L: brain lysate**
**AB: antibody**
**IP: immunoprecipitate**
**IB: immunoblot**
**SUP: supernatant**
**WT: wildtype**
**FKO: functional knockout**
**HPC: hippocampus**

**Odyssey CLx Imager using Image Studio software (Ver 5.2) (LI-COR Biosciences) was used to capture all images**

# S1 Fig-2

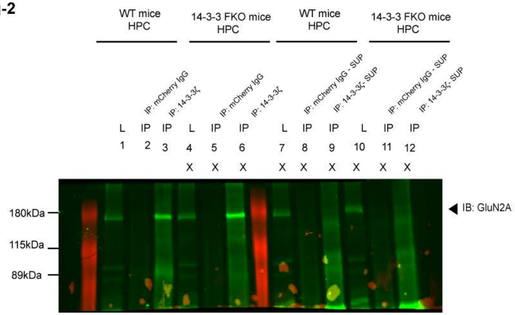


**raw file**

**Same blot from S2 Fig-1**
**Stripped and reprobed with GluN2A**

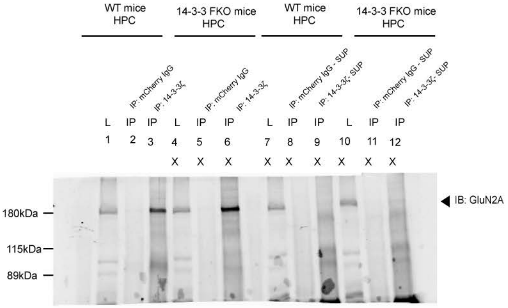


**Inverted image (grayscale)**

**L: brain lysate**
**AB: antibody**
**IP: immunoprecipitate**
**IB: immunoblot**
**SUP: supernatant**
**WT: wildtype**
**FKO: functional knockout**
**HPC: hippocampus**

**Odyssey CLx Imager using Image Studio software (Ver 5.2) (LI-COR Biosciences) was used to capture all images**

**S1 Fig-3**

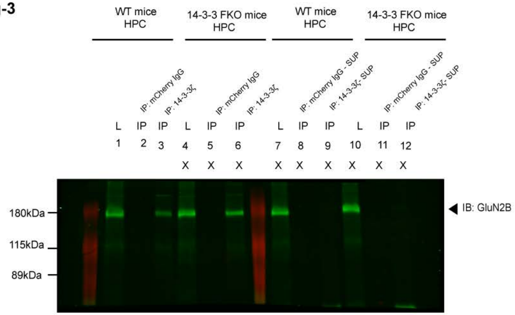


**raw file**

**Same blot from S2 Fig-1 and 2**
**Stripped and reprobed with GluN2B**

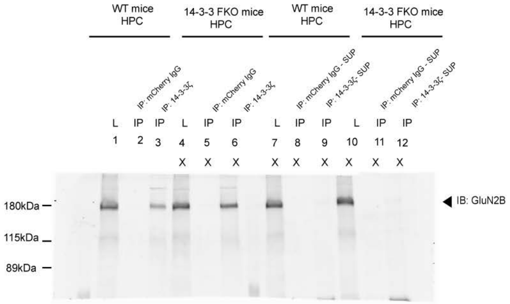


**Inverted image (grayscale)**

**L: brain lysate**
**AB: antibody**
**IP: immunoprecipitate**
**IB: immunoblot**
**SUP: supernatant**
**WT: wildtype**
**FKO: functional knockout**
**HPC: hippocampus**

**Odyssey CLx Imager using Image Studio software (Ver 5.2) (LI-COR Biosciences) was used to capture all images**

**S1 Fig-4**

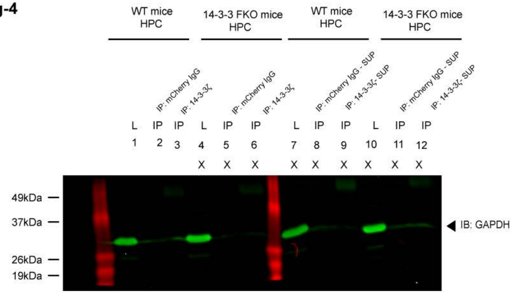


**raw file**

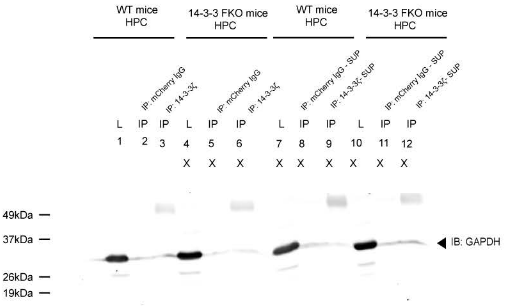


**Inverted image (grayscale)**

**L: brain lysate**
**AB: antibody**
**IP: immunoprecipitate**
**IB: immunoblot**
**SUP: supernatant**
**WT: wildtype**
**FKO: functional knockout**
**HPC: hippocampus**

**Odyssey CLx Imager using Image Studio software (Ver 5.2) (LI-COR Biosciences) was used to capture all images**

## S1 Fig-5

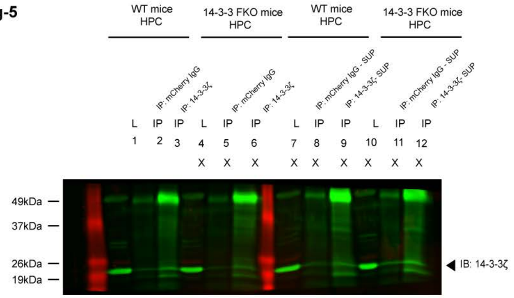


**raw file**

**Same blot from S1 Fig-4**
**Stripped and reprobed with 14-3-3 zeta**

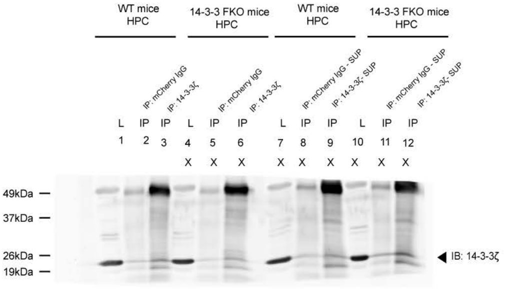


**Inverted image (grayscale)**

**L: brain lysate**
**AB: antibody**
**IP: immunoprecipitate**
**IB: immunoblot**
**SUP: supernatant**
**WT: wildtype**
**FKO: functional knockout**
**HPC: hippocampus**

**Odyssey CLx Imager using Image Studio software (Ver 5.2) (LI-COR Biosciences) was used to capture all images**

## S2 Fig B-1

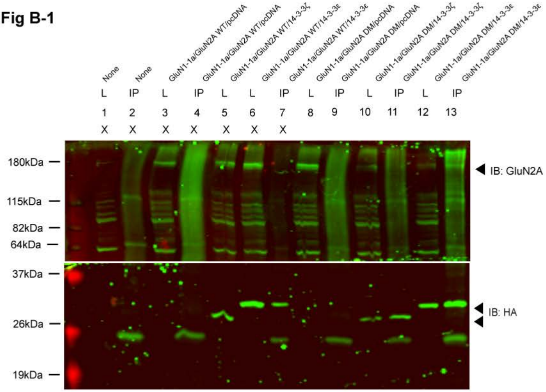


**raw file**

**Same blot cut below 64kDa**
**Top blot probed with GluN2A**
**Bottom blot probed with HA**

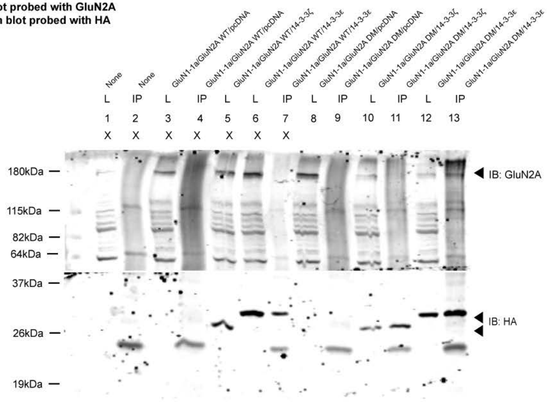


**Inverted image (grayscale)**

**L: cell lysate**
**IP: immunoprecipitate**
**IB: immunoblot**
**WT: wildtype**
**DM: double mutant**

**Odyssey CLx Imager using Image Studio software (Ver 5.2) (LI-COR Biosciences) was used to capture all images**

## S2 Fig B-2

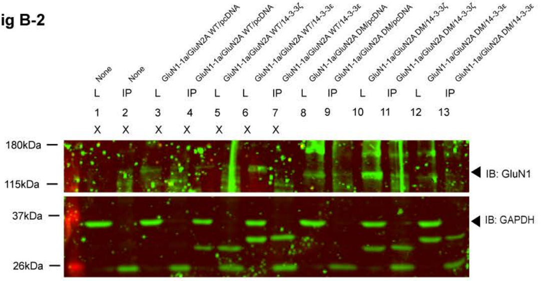


**raw file**

**Same blot from S2 Fig B-1**
**Stripped and reprobed**
**Top blot probed with GluN1**
**Bottom blot probed with GAPDH**

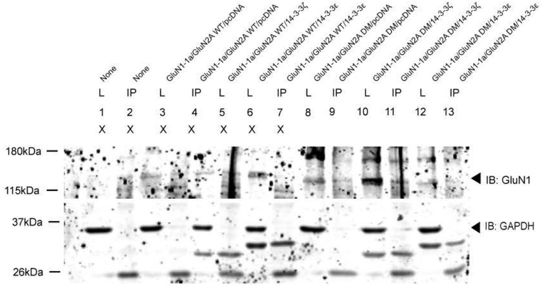


**Inverted image (grayscale)**

**Odyssey CLx Imager using Image Studio software (Ver 5.2) (LI-COR Biosciences) was used to capture all images**

**L: cell lysate**
**IP: immunoprecipitate**
**IB: immunoblot**
**WT: wildtype**
**DM: double mutant**

## S2 Fig C-1

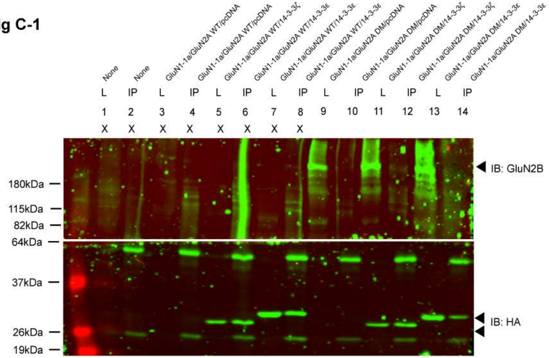


raw file

Same blot cut below 64kDa
Top blot probed with GluN2B
Bottom blot probed with HA

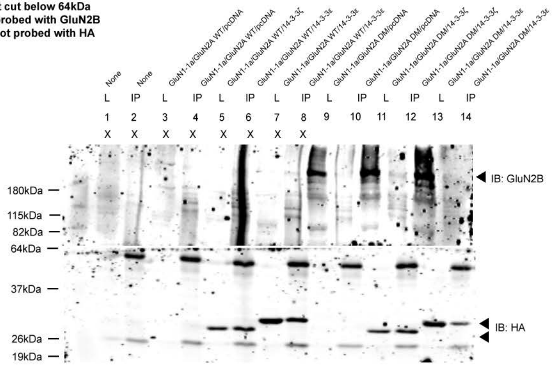


Inverted image (grayscale)

Odyssey CLx Imager using Image Studio software (Ver 5.2) (LI-COR Biosciences) was used to capture all images

L: cell lysate
IP: immunoprecipitate
IB: immunoblot
WT: wildtype
DM: double mutant

## S2 Fig C-2

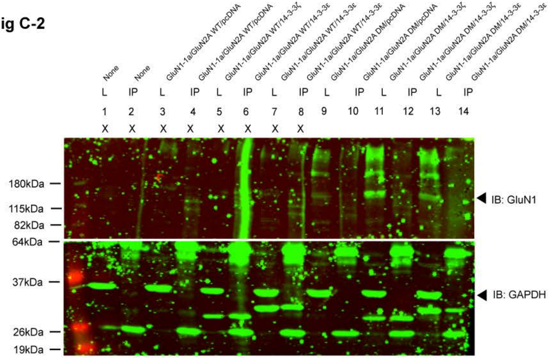


raw file

**Same blot as S2 Fig C-1**
**Stripped and reprobed;**
**Top blot probed with GluN1**
**Bottom blot probed with GAPDH**

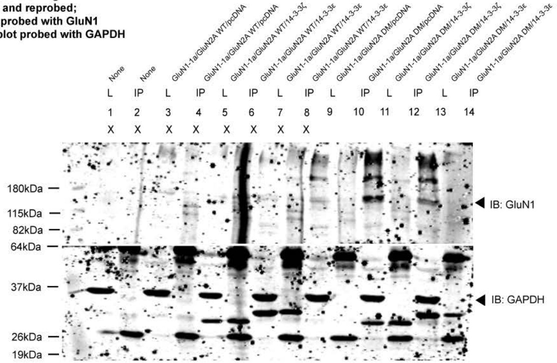


Inverted image (grayscale)

**L: cell lysate**
**IP: immunoprecipitate**
**IB: immunoblot**
**WT: wildtype**
**DM: double mutant**

**Odyssey CLx Imager using Image Studio software (Ver 5.2) (LI-COR Biosciences) was used to capture all images**
